# Alzheimer Disease Blood Biomarkers in Patients With Out-of-Hospital Cardiac Arrest

**DOI:** 10.1001/jamaneurol.2023.0050

**Published:** 2023-03-06

**Authors:** Nicholas J. Ashton, Marion Moseby-Knappe, Andrea L. Benedet, Lana Grötschel, Juan Lantero-Rodriguez, Thomas K. Karikari, Christian Hassager, Matt P. Wise, Pascal Stammet, Jesper Kjaergaard, Hans Friberg, Niklas Nielsen, Tobias Cronberg, Henrik Zetterberg, Kaj Blennow

**Affiliations:** 1 Department of Psychiatry and Neurochemistry, Institute of Neuroscience & Physiology, The Sahlgrenska Academy at the University of Gothenburg, Mölndal, Sweden; 2 Wallenberg Centre for Molecular and Translational Medicine, University of Gothenburg, Gothenburg, Sweden; 3 Institute of Psychiatry, Psychology and Neuroscience, Maurice Wohl Clinical Neuroscience Institute, King’s College London, London, United Kingdom; 4 National Institute for Health and Care Research Biomedical Research Centre for Mental Health and Biomedical Research Unit for Dementia, South London and Maudsley National Health Service Foundation, London, United Kingdom; 5 Skåne University Hospital, Department of Clinical Sciences, Neurology, Lund University, Lund, Sweden; 6 McGill Centre for Studies in Aging, Translational Neuroimaging Laboratory, McGill University, Montreal, Quebec, Canada; 7 Department of Cardiology, The Heart Centre, Rigshospitalet, Copenhagen University Hospital, Copenhagen, Denmark; 8 Adult Critical Care, University Hospital of Wales, Heath Park, Cardiff, United Kingdom; 9 Department of Anesthesia and Intensive Care, Centre Hospitalier de Luxembourg, Luxembourg, Luxembourg; 10 Faculty of Science, Technology and Medicine, Department of Life Sciences and Medicine, University of Luxembourg, Esch-sur-Alzette, Luxembourg; 11 Department of Clinical Sciences Lund, Anesthesia & Intensive Care Section, Helsingborg Hospital, Lund University, Lund, Sweden; 12 Clinical Neurochemistry Laboratory, Sahlgrenska University Hospital, Mölndal, Sweden; 13 Department of Neurodegenerative Disease, University College London Queen Square Institute of Neurology, London, United Kingdom; 14 United Kingdom Dementia Research Institute at University College London, University College London, London, United Kingdom; 15 Hong Kong Center for Neurodegenerative Diseases, Hong Kong, China

## Abstract

**Importance:**

Blood phosphorylated tau (p-tau) and amyloid-β peptides (Aβ) are promising peripheral biomarkers of Alzheimer disease (AD) pathology. However, their potential alterations due to alternative mechanisms, such as hypoxia in patients resuscitated from cardiac arrest, are not known.

**Objective:**

To evaluate whether the levels and trajectories of blood p-tau, Aβ42, and Aβ40 following cardiac arrest, in comparison with neural injury markers neurofilament light (NfL) and total tau (t-tau), can be used for neurological prognostication following cardiac arrest.

**Design, Setting, and Participants:**

This prospective clinical biobank study used data from the randomized Target Temperature Management After Out-of-Hospital Cardiac Arrest (TTM) trial. Unconscious patients with cardiac arrest of presumed cardiac origin were included between November 11, 2010, and January 10, 2013, from 29 international sites. Serum analysis for serum NfL and t-tau were performed between August 1 and August 23, 2017. Serum p-tau, Aβ42, and Aβ40 were analyzed between July 1 and July 15, 2021, and between May 13 and May 25, 2022. A total of 717 participants from the TTM cohort were examined: an initial discovery subset (n = 80) and a validation subset. Both subsets were evenly distributed for good and poor neurological outcome after cardiac arrest.

**Exposures:**

Serum p-tau, Aβ42, and Aβ40 concentrations using single molecule array technology. Serum levels of NfL and t-tau were included as comparators.

**Main Outcomes and Measures:**

Blood biomarker levels at 24 hours, 48 hours, and 72 hours after cardiac arrest. Poor neurologic outcome at 6-month follow-up, defined according to the cerebral performance category scale as category 3 (severe cerebral disability), 4 (coma), or 5 (brain death).

**Results:**

This study included 717 participants (137 [19.1%] female and 580 male [80.9%]; mean [SD] age, 63.9 [13.5] years) who experienced out-of-hospital cardiac arrest. Significantly elevated serum p-tau levels were observed at 24 hours, 48 hours, and 72 hours in cardiac arrest patients with poor neurological outcome. The magnitude and prognostication of the change was greater at 24 hours (area under the receiver operating characteristic curve [AUC], 0.96; 95% CI, 0.95-0.97), which was similar to NfL (AUC, 0.94; 95% CI, 0.92-0.96). However, at later time points, p-tau levels decreased and were weakly associated with neurological outcome. In contrast, NfL and t-tau maintained high diagnostic accuracies, even 72 hours after cardiac arrest. Serum Aβ42 and Aβ40 concentrations increased over time in most patients but were only weakly associated with neurological outcome.

**Conclusions and Relevance:**

In this case-control study, blood biomarkers indicative of AD pathology demonstrated different dynamics of change after cardiac arrest. The increase of p-tau at 24 hours after cardiac arrest suggests a rapid secretion from the interstitial fluid following hypoxic-ischemic brain injury rather than ongoing neuronal injury like NfL or t-tau. In contrast, delayed increases of Aβ peptides after cardiac arrest indicate activation of amyloidogenic processing in response to ischemia.

## Key Points

**Question:**

Are Alzheimer disease blood biomarkers altered in patients with hypoxic-ischemic injury after cardiac arrest?

**Findings:**

In this case-control study, blood phosphorylated tau (p-tau) levels were significantly elevated 24 hours after cardiac arrest and were associated with poorer neurologic outcome. Unlike neurofilament light (NfL) and total tau (t-tau), p-tau levels decreased after 24 hours and were no longer associated with neurological outcome; amyloid-β (Aβ) peptides demonstrated moderate increases and were only weakly associated with neurological outcome.

**Meaning:**

These findings of the increase and dynamics of p-tau in blood following cardiac arrest, which differ from those of t-tau and NfL, suggest alternative mechanisms of p-tau origin and release from the increases observed due to Aβ deposition and neuronal tau phosphorylation in Alzheimer disease.

## Teaser

This case-control study evaluates the potential role of blood phosphorylated tau and amyloid-β peptides in neurological prognostication following cardiac arrest.

## Introduction

Extracellular amyloid-β (Aβ) plaques and neurofibrillary tangles composed of phosphorylated tau (p-tau) are the key protein signatures of a brain with Alzheimer disease (AD). Aβ peptides (Aβ42 and Aβ40) and p-tau can be quantified in cerebrospinal fluid (CSF) and, more recently, in blood, where both biomarkers show high accuracy in determining underlying AD brain pathology^1^ in both symptomatic^2,3^ and asymptomatic disease.^4,5^ When using these biomarkers in studies focusing on disease mechanisms or treatment effects, it is important to understand the differences between biomarker changes observed in CSF and blood relating to the proposed underlying pathophysiology. In this context, it should be acknowledged that findings in biomarker studies, such as increases of p-tau with Aβ position emission tomography ligand retention,^6^ are only associations and do not necessarily imply causal relationships. The study of candidate biomarkers in disorders unrelated to the primary disease pathology may give important and novel insights into mechanisms for their change and associations with brain pathophysiology. For example, across the AD continuum, in both cognitively unimpaired and impaired elderly adults, high associations between CSF levels of p-tau and total tau (t-tau) are observed, suggesting these biomarkers reflect AD- and aging-related changes in tau homeostasis. However, in poststroke patients, CSF t-tau increases in association with stroke severity, while p-tau does not.^7^ This strengthens the view that p-tau is a marker for AD-type tau pathology, while t-tau reflects neuronal injury, a notion later supported by associations between p-tau and tau positron emission tomography signal.^8^

In addition to AD and other neurodegenerative disorders, blood biomarkers are also warranted in prognostication in unconscious patients experiencing hypoxic-ischemic brain injury following cardiac arrest. Current guidelines recommend a multimodal approach in the prediction of outcome after cardiac arrest,^9^ and using blood biomarkers has shown promise as a simple and rapid assessment of brain injury.^10^ There is overlap between putative blood biomarkers for neurological outcome after cardiac arrest and neurodegenerative processes in aging disorders,^11^ namely neurofilament light (NfL),^10,12^ t-tau,^13,14^ and glial fibrillary acidic protein.^15,16^ Interestingly, except for widely reported increases in AD,^1^ blood levels of p-tau also show a marked increase acutely following mild traumatic brain injury.^17^ However, blood biomarkers most reflective of AD pathophysiology, p-tau and Aβ peptides, have not, to our knowledge, been investigated in the initial stages in patients who have experienced out-of-hospital cardiac arrest.

In the present study, we evaluated whether serum p-tau, Aβ42, Aβ40, and Aβ42/40 ratio, the most specific biomarkers for AD pathophysiology in blood, had altered levels after cardiac arrest. Furthermore, we examined if these biomarkers were associated with the severity of neurological outcome and compared their longitudinal trajectories (24 to 72 hours after cardiac arrest) to biomarkers of brain injury after cardiac arrest, namely NfL and t-tau.

## Methods

### Study Cohort, Design, and Outcome

This study is a targeted analysis of serum p-tau181, Aβ42, and Aβ40 concentrations in participants from the international multicenter Target Temperature Management After Out-of-Hospital Cardiac Arrest (TTM) trial. The trial recruited unconscious (Glasgow Coma Scale score less than 8) adult patients (18 years and older) with out-of-hospital cardiac arrest of presumed cardiac origin from November 11, 2010, to January 10, 2013.^18^ Blood samples were collected prospectively at 24, 48, and 72 hours after cardiac arrest. Serum analysis for the blood biomarkers t-tau and NfL were analyzed between August 1 and August 23, 2017, as previously published.^14,19^ Written informed consent was waived or obtained from all patients or relatives in line with the Declaration of Helsinki^20^ and according to the national legislation relevant to each participating site. The trial protocols were approved by ethical committees in each participating country. The Standards for Reporting of Diagnostic Accuracy Studies guidelines were followed.^21^

To allow for optimal assessment of possible changes in blood levels of p-tau and Aβ, we initially selected 80 participants (discovery cohort) from the TTM trial evenly distributed between good neurological outcome (Cerebral Performance Category scale score 2 or less) and poor neurological outcome (Cerebral Performance Category scale score 3 or greater). Good and poor outcome groups were further divided by low and high levels of serum NfL as stipulated in eTable 1 in Supplement 1.

The validation cohort was an unselected sample encompassing the full TTM trial, consisting of 717 participants. In the validation cohort, 357 participants had a good neurological outcome and 360 had a poor neurological outcome (Table).

**Table.** Demographic Characteristics and Prognostic Accuracy of Blood Biomarkers at 24, 48, and 72 Hours After Cardiac Arrest to Predict Neurologic Outcome at 6 Months

	Total AUC (95% CI)	Outcome, median (IQR), pg/mL		*P* value
		Good^a^	Poor^b^	
No.	NA	357	360	
Age, mean (SD), y	NA	59.9 (12.3)	68.0 (14.0)	<.001
Female, No. (%)	NA	56 (15.7)	81 (22.5)	.02
Male, No. (%)	NA	301 (84.3)	279 (77.5)	.02
**24 h after cardiac arrest**				
p-Tau 181	0.96 (0.95-0.97)	6.10 (3.50)	24.30 (36.10)	<.001
Aβ40	0.50 (0.45-0.55)	108 (114)	105 (147)	.10
Aβ42	0.49 (0.44-0.54)	5.56 (4.29)	5.39 (5.73)	.60
Aβ42/40	0.54 (0.49-0.59)	0.053 (0.027)	0.058 (0.049)	.20
NfL	0.94 (0.93-0.96)	37.1 (50.0)	1430.0 (3280.0)	<.001
t-Tau	0.80 (0.77-0.84)	2.40 (3.85)	11.90 (39.50)	<.001
Creatinine	NA	0.780 (0.360)	1.12 (0.963)	<.001
**48 h after cardiac arrest**				
p-Tau181	0.68 (0.63-0.73)	5.83 (3.83)	8.54 (7.11)	<.001
Aβ40	0.56 (0.51-0.61)	142 (130)	169 (164)	.40
Aβ42	0.56 (0.50-0.61)	7.56 (5.85)	8.46 (6.85)	.01
Aβ42/40	0.51 (0.46-0.56)	0.056 (0.023)	0.054 (0.026)	.06
NfL	0.93 (0.91-0.95)	46.0 (75.3)	3240.0 (7650.0)	<.001
t-Tau	0.89 (0.86-0.92)	1.86 (2.26)	45.70 (330.00)	<.001
Creatinine	NA	0.880 (0.450)	1.330 (1.080)	<.001
**72 h after cardiac arrest**				
p-Tau181	0.60 (0.55-0.65)	6.22 (4.32)	7.16 (5.31)	<.001
Aβ40	0.59 (0.54-0.64)	167 (135)	207 (203)	.01
Aβ42	0.60 (0.55-0.65)	8.18 (4.85)	9.48 (8.02)	<.001
Aβ42/40	0.49 (0.44-0.55)	0.050 (0.018)	0.050 (0.018)	.50
NfL	0.94 (0.92-0.95)	53.6 (91.4)	3410.0 (6970.0)	<.001
t-Tau	0.89 (0.86-0.92)	1.50 (1.63)	38.4 (239.0)	<.001
Creatinine	NA	1.01 (0.56)	1.57 (1.11)	<.001

Abbreviations: Aβ40, amyloid-β 40; Aβ42, amyloid-β 42; AUC, area under the receiver operating characteristic curve; NfL, neurofilament light chain; p-tau181, tau phosphorylated at threonine 181; t-tau, total tau.

^a^ Defined as Cerebral Performance Category scale score 2 or lower at 6 months after cardiac arrest.

^b^ Defined as Cerebral Performance Category scale score 3 or higher at 6 months after cardiac arrest.

### Blood Collection and Biomarker Analysis

All serum biomarker analyses were performed after completion of the TTM-trial.^18^ Serum NfL and t-tau concentration had been measured previously using an in-house NfL assay on the Simoa HD-1 platform^14,19^ and a commercially available Simoa assay (Quanterix), respectively. Serum Aβ40 and Aβ42 concentrations were measured by the commercially available Neurology 3-Plex A Advantage Kit (Quanterix). Serum p-tau181 concentrations were measured using the in-house method from the University of Gothenburg.^22^ For the discovery cohort, serum p-tau and Aβ were analyzed between July 1 and July 15, 2021. For the validation cohort, serum p-tau and Aβ were analyzed between May 13 and May 25, 2022.

### Statistical Analysis

All statistical tests were performed using R version 3.6.3 (R Foundation). For demographic comparisons of categorical variables, χ^2^ tests were used while *t* tests were used for continuous variables. Spearman rank tests evaluated the correlation between biomarkers at specific time points. Fold changes were calculated using biomarker concentrations of the good outcome group at 24 hours as reference and the data was plotted using locally estimated scatterplot smoothing. Between each outcome group, *t* test was used to compare biomarker levels and fold changes. Receiver operating characteristic curve analysis assessed the accuracy of neurological outcome (good vs poor) based on biomarkers concentrations at each time point. Receiver operating characteristic curve 95% CIs of sensitivities and specificities were also computed (Youden index), and a smoothing parameter was applied in the graphic output. Longitudinal biomarker trajectories were compared between groups using linear mixed-effect models, where the biomarkers were set as continuous dependent variables and the interaction between neurological outcome groups (good and poor) and time (hours after event) was the independent variable. The models considered random intercepts. Biomarkers with nonnormal distribution (based on visual inspection of histograms) were log10 transformed prior to parametric analyses. As a sensitivity analysis, the procedures described above were repeated including only participants and visits with no missing biofluid data. All tests were 2-sided, and *P* less than .05 was considered statistically significant.

## Results

### Participant Characteristics

Of 80 patients in the discovery cohort, 11 (13.75%) were female and 69 (86.25%) were male. The mean (SD) age was 60.8 (12) years. Patients were significantly older in the poor neurologic outcome group (mean [SD] age, 62.8 [12.9] years) compared with the good neurological outcome group (mean [SD] age, 58.8 [11.1] years) (*P* = .04). The discovery cohort was further characterized by retrospective measurements of NfL at 72 hours, indicating the degree of ongoing neural injury. In the good neurologic outcome group, the high NfL group was significantly older than the low NfL group. In the poor neurologic outcome group, there was no significant difference in age observed between the high NfL group and the low NfL group.

The validation cohort consisted of 717 patients with cardiac arrest (137 [19.1%] female and 580 [80.9%] male; mean [SD] age, 63.9 [13.5] years; 357 [49.8%] with good neurologic outcome and 360 [50.2%] with poor neurologic outcome) (Table). This cohort was not preselected by previous serum biomarker results to allow for the comparison of other biomarkers and NfL. Once more, the poor neurologic outcome group was significantly older than the good neurologic outcome group (mean [SD] age, 68.0 [14.0] years vs 59.9 [12.3] years, respectively; *P* < .001), and participants were predominantly male.

### Serum p-Tau, Aβ40, and Aβ42 Changes in Cardiac Arrest: Discovery Cohort

In the discovery cohort, 24 hours after cardiac arrest, serum p-tau was significantly elevated only in patients with poor neurological outcome (eTable 2 in Supplement 1) with a good diagnostic accuracy of neurological outcome (area under the receiver operating characteristic curve [AUC], 0.75; 95% CI, 0.64-0.88) (eFigure 1 and eTable 2 in Supplement 1). At later time points, significant elevations in the poor outcome group were also observed (eFigure 1 and eTable 2 in Supplement 1) but with significantly lower prognostic accuracy of neurological outcome. The serum levels of Aβ40 and Aβ42 increased in most patients over time but did not differ significantly between neurological outcome groups at 24 hours, despite being higher in the poor neurologic outcome group (eFigure 1 and eTable 2 in Supplement 1). At 48 hours and 72 hours, both Aβ peptides were significantly higher in the poor neurologic outcome group (eTable 2 in Supplement 1). Serum Aβ40 and Aβ42 were correlated (ρ, 0.79; *P* < .001) and the Aβ42/Aβ40 ratio did not significantly change between outcome groups at any time point, despite being lower in the poor neurologic outcome group. The longitudinal trajectories of p-tau, Aβ40, Aβ42, and Aβ42/40 ratio from 24 hours to 72 hours in the discovery cohort are shown in eFigure 2 in Supplement 1.

### A Replication of Serum p-Tau Changes After Cardiac Arrest: Trajectories Over Time in Comparison to NfL and t-Tau

**Figure 1.**
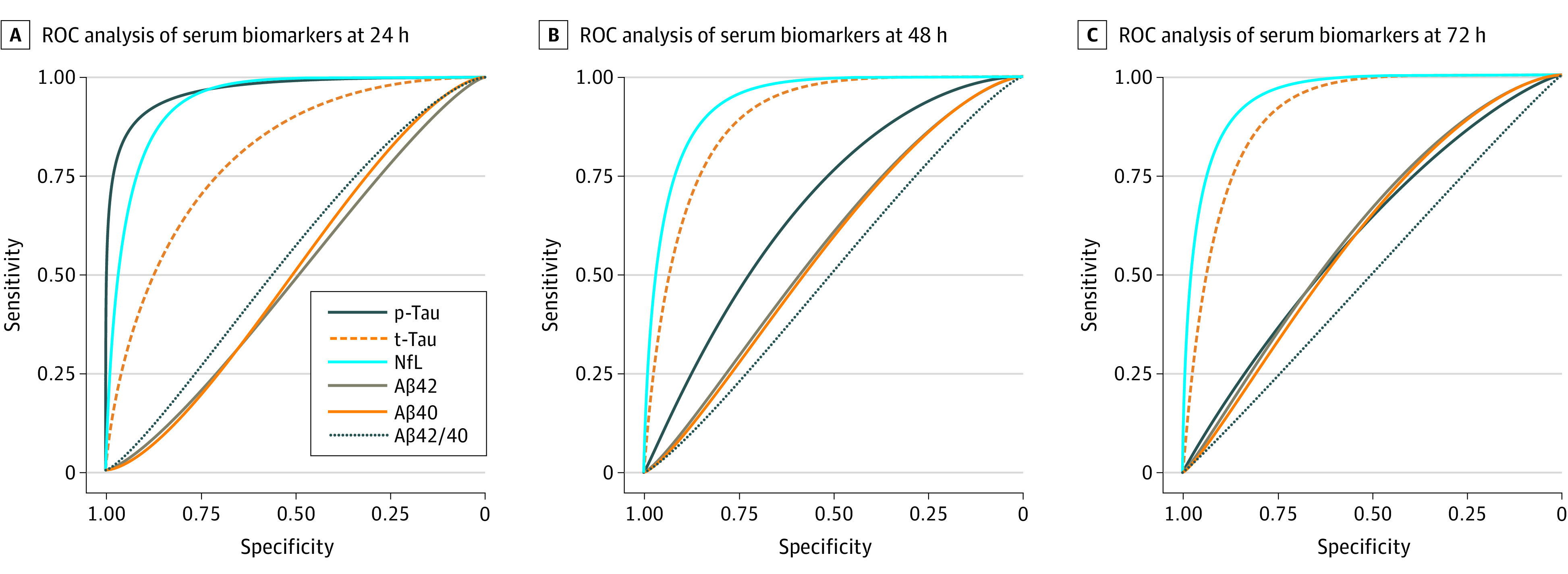
Biomarker Discriminative Accuracy Over Time Receiver operating characteristic curve (ROC) analysis was performed to test the accuracy of serum biomarkers to discriminate between outcome groups at 24 hours, 48 hours, and 72 hours. Aβ40 indicates amyloid-β 40; Aβ42, amyloid-β 42; NfL, neurofilament light; p-tau, phosphorylated tau; t-tau, total tau.

In the larger and unselected validation cohort, we again observed significantly increased p-tau levels in patients with poor neurologic outcome at 24 hours, 48 hours, and 72 hours (Table; eFigure 3 in Supplement 1). No significant change in p-tau levels were observed in the good neurological outcome group between 24-72 hours (eFigure 3 in Supplement 1). At 24 hours, serum p-tau demonstrated high prognostic value to determine neurologic outcome at 6 months (AUC, 0.96; 95% CI, 0.95-0.97) (Figure 1A), which was not significantly different from serum NfL (AUC, 0.94; 95% CI, 0.92-0.96) but significantly better than t-tau (AUC, 0.80; 95% CI, 0.77-0.84; *P* < .001). Yet the prognostic capabilities of p-tau were significantly lower than NfL and t-tau at 48 hours (AUC for p-tau, 0.68; 95% CI, 0.63-0.73 vs AUC for NfL, 0.94; 95% CI, 0.91-0.95 and AUC for t-tau, 0.89; 95% CI, 0.86-0.92) (Table; Figure 1B) and 72 hours (AUC for p-tau, 0.60; 95% CI, 0.55-0.65 vs AUC for NfL, 0.94; 95% CI, 0.92-0.95 and AUC for t-tau, 0.89; 95% CI, 0.86-0.92) (Table; Figure 1C).

Aβ40, Aβ42, and the Aβ42/40 ratio did not significantly change between groups at 24 hours; however, a significant difference was observed at 48 hours for Aβ42 and both Aβ peptides at 72 hours. Thus, the diagnostic accuracy of Aβ remained to low at all time points (Table; Figure 1).

**Figure 2.**
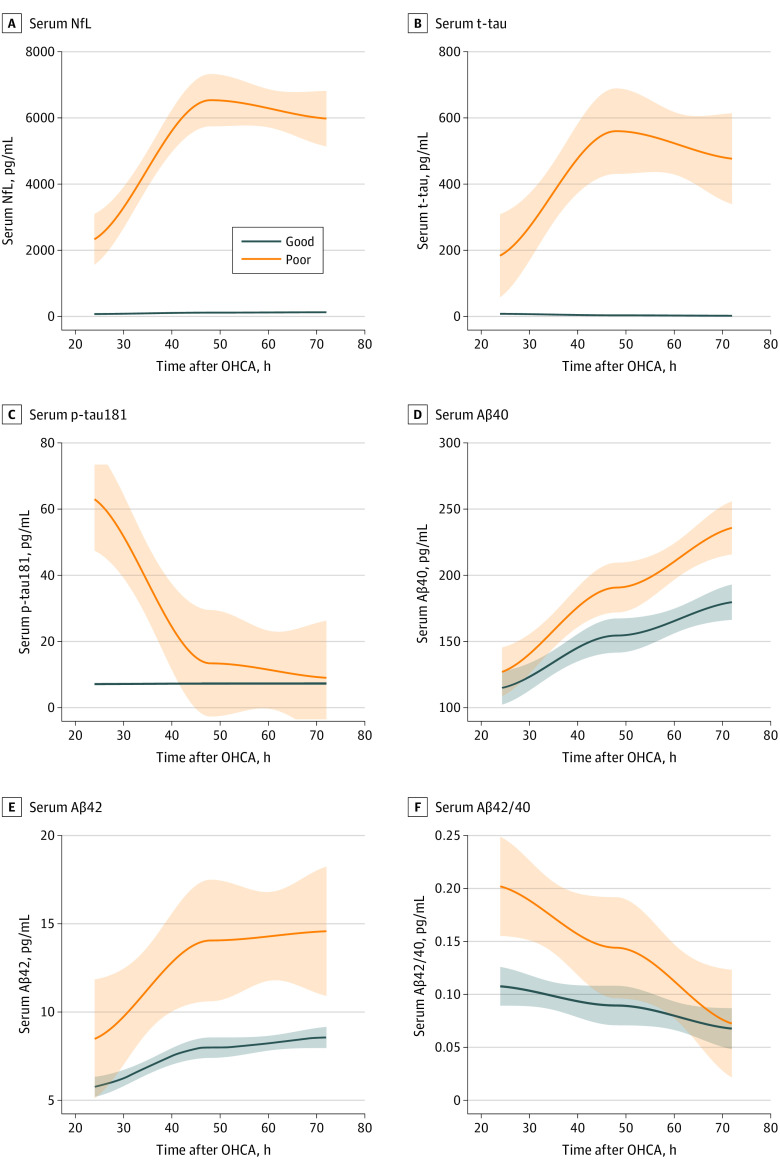
Biomarker Changes Over Time After 24 Hours in the Validation Cohort Locally estimated scatterplot smoothing plots showing the mean change in biomarker levels over time: linear mixed-effect models analysis indicated that, except for Aβ42/40, the trajectory of the biomarkers differs between good and poor outcome groups (*P* < .001). The solid lines indicate the regression line; the shaded area, 95% CIs. Aβ40 indicates amyloid-β 40; Aβ42, amyloid-β 42; NfL, neurofilament light; OHCA, out-of-hospital cardiac arrest; p-tau181, tau phosphorylated at threonine 181; t-tau, total tau.

As expected, different longitudinal trajectories of p-tau in comparison to NfL and t-tau were observed in the poor neurologic outcome group (Figure 2). While p-tau was highest at 24 hours, NfL and t-tau reached peak levels at 48 hours and were largely maintained at 72 hours. In contrast, at 72 hours, p-tau levels in the poor neurologic outcome group were approaching levels of good neurologic outcome. Linear mixed-effect models confirmed that the biomarkers’ mean rate of change in the poor outcome group was different from the rate of change of the good outcome group between 24 hours and 72 hours (summary statistics reported in eTable 3 in Supplement 1).

### Magnitude Differences Between Biomarkers After Cardiac Arrest

**Figure 3.**
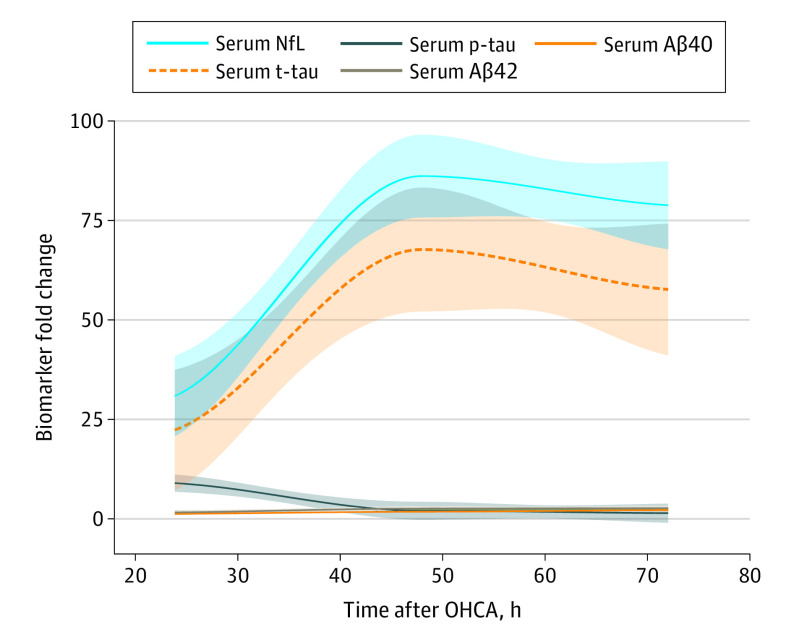
Fold Changes of Blood Levels of p-Tau, NfL, t-Tau, Aβ40, Aβ42, and Aβ42/40 Mean biomarker fold changes over time in the poor outcome group plotted using the locally estimated scatterplot smoothing method. Fold changes were calculated based on the mean biomarker levels of the good outcome group at 24 hours. The solid lines indicate the regression line; the shaded area, 95% CIs. Aβ40 indicates amyloid-β 40; Aβ42, amyloid-β 42; NfL, neurofilament light chain; p-tau, phosphorylated tau; t-tau, total tau.

In comparing the fold changes of all biomarkers in the poor neurologic outcome group to those in the good neurologic outcome group (Figure 3; eTable 4 in Supplement 1), a substantially larger mean (SD) fold change of NfL (30.8 [36.8]) and t-tau (22.8 [81.8]) was observed at 24 hours in comparison to p-tau (8.8 [29.8]) despite similar prognostic performance. In line with the prognostic characteristics, the mean (SD) fold changes of NfL and t-tau increased substantially at 48 hours (86.2 [121.0] and 67.7 [166.0], respectively) (Figure 3) and 72 hours (78.8 [110.0] and 57.6 [170.0], respectively) (Figure 3) after cardiac arrest in the poor outcome group while mean (SD) p-tau fold changes were lower (1.9 [2.6] at 48 hours and 1.3 [0.8] at 72 hours). Serum Aβ40 and Aβ42 fold changes were small in comparison (Figure 3; eTable 4 in Supplement 1). The fold change of Aβ40 in the poor neurologic outcome group did not exceed 2 at any time point, whereas the fold change of Aβ42 peaked at 2.5 at 72 hours.

### Correlation Between Blood Biomarkers in Cardiac Arrest

Serum p-tau and NfL were correlated at 24 hours (*r* = 0.67; *P* < .001), and the correlations became gradually weaker at 48 hours (*r* = 0.33; *P* < .001) and 72 hours (*r* = 0.19 *P* < .001) (eFigure 4C in Supplement 1). The correlations between p-tau and t-tau followed a similar trajectory (24 hours, *r* = 0.65; *P* < .001; 48 hours, *r* = 0.4; *P* < .001; 72 hours, *r* = 0.22; *P* < .001). The correlation of NfL and t-tau strengthened between 24 and 72 hours. There was no significant correlation between Aβ peptides and other biomarkers at 24 hours, with weak but significant correlations between 48 and 72 hours. Creatinine was significantly correlated with p-tau (*r* = 0.51; *P* < .001), t-tau (*r* = 0.41; *P* < .001), and NfL (*r* = 0.41; *P* < .001) at 24 hours, which gradually weakened. In contrast, Aβ and creatinine had a strengthening of correlation, which was strongest at 72 hours (Aβ42, *r* = 0.43; *P* < .001; Aβ40, *r* = 0.40; *P* < .001). The was no correlation between the Aβ ratio and creatinine at any time point.

The correlations in delta change (∆) of all biomarkers are shown in eFigure 5 in Supplement 1. The inverse correlation between ∆p-tau and ∆NfL was stronger (*r* = −0.57; *P* < .001) than ∆p-tau and ∆t-tau (*r* = −0.20; *P* < .001). The ∆creatinine was not correlated with ∆p-tau but was significantly correlated with ∆Aβ42 (*r* = 0.35; *P* < .001), ∆Aβ40 (*r* = 0.32; *P* < .001), ∆NfL (*r* = 0.22; *P* < .001), and ∆t-tau (*r* = 0.16; *P* < .001).

## Discussion

In this case-control study, we examined the central AD blood biomarkers, p-tau and Aβ, in patients with severe hypoxic-ischemic brain injury due to cardiac arrest. The key results demonstrate a rapid increase of p-tau into the bloodstream within 24 hours following cardiac arrest. However, this increase was highly specific to individuals exhibiting a poor neurologic outcome; individuals with a good neurological outcome exhibited minimal change. Results in the larger validation cohort showed a prognostic value similar to NfL and higher than t-tau at 24 hours; however, the direct magnitude changes of t-tau and NfL were shown to be far larger than p-tau. At 48-72 hours after cardiac arrest, p-tau levels in patients with poor neurological outcome largely decreased and approached the concentrations of patients with good neurological outcome, which remained at a normal level during this acute period.

Blood p-tau is a meaningful and highly specific measure of underlying AD neuropathology^23^ with imminent therapeutic and diagnostic application.^24^ To our knowledge, this is the first account of p-tau levels in patients with cardiac arrest. The mechanisms that promote increased p-tau in the bloodstream likely differ between these 2 conditions. In AD, p-tau in blood is expected to be a continual secretion in response to extracellular Aβ plaques and intracellular tau tangles, pathologies that develop over many decades and are unlikely to be ubiquitously present in the age group of patients included in this study. In contrast, after cardiac arrest, a transient opening of the blood-brain barrier,^25^ in more severe cases, likely causes a direct efflux of central nervous system–specific peptides from the interstitial space of the brain into the bloodstream or via the glymphatic system.^26^ This is seemingly no different for p-tau, and we found high levels at 24 hours, showing high accuracy in the validation sample to predict neurological outcome. At this time point, the prognostic capabilities of p-tau were comparable to those of NfL and t-tau.^16^ It must be noted that the magnitude of this change was noticeably lower for p-tau, likely demonstrating the lower availability of p-tau in the interstitial fluid in comparison to NfL and t-tau, which demonstrated much larger fold changes after cardiac arrest. However, the high predictive accuracy of p-tau quickly deteriorated, whereas t-tau and NfL maintained their high predictive value even at 72 hours after cardiac arrest. Our data supports the notion that, in the immediate response to cardiac arrest, p-tau, NfL, and t-tau initially reflect a similar mechanistic process that, in more severe cases, disrupts the blood-brain barrier and allows for the leakage of central nervous system proteins into the bloodstream. In addition to high prognostic accuracy, this viewpoint is supported by the high and similar correlations of p-tau, t-tau, and NfL at 24 hours, which was substantially higher than what is reported in AD studies.^27^ At 48 hours and 72 hours, however, the association of p-tau with NfL and t-tau was markedly reduced, p-tau levels were lower, and the prognostic capabilities of neurological outcome for p-tau were poor. At the same time, the NfL and t-tau correlation was sustained, demonstrating that p-tau was rapidly cleared from the bloodstream after the initial insult but did not continue to secrete into the bloodstream at a high-rate. Thus, in this context, p-tau does not appear to reflect ongoing neural injury but rather serves as an indicator of the severity of the initial insult, while NfL and t-tau levels continued to increase in concentration, maintaining prognostic accuracy and reflecting ongoing central nervous system damage.

We also found increasing blood levels of both Aβ40 and Aβ42, which were significantly different in the poor neurologic outcome group at 48 hours (Aβ42) and 72 hours (Aβ40 and Aβ42) but not at 24 hours. Visually, the temporal trajectories of Aβ42 followed a similar pattern to NfL and t-tau, albeit on a substantially lower magnitude. The Aβ42/Aβ40 ratio did not change significantly. These results support our previous pilot study showing a more than 3-fold increase in serum Aβ42 acutely after cardiac arrest and correlating with clinical outcome.^28^ The pathophysiological interpretation of such changes in Aβ metabolism or release is also unclear. Increasing results support that brain hypoxia-ischemia may increase amyloidogenic processing of amyloid precursor protein with activation of the β-secretase and γ-secretase pathways and production of Aβ peptides.^29^ This change in amyloid precursor protein metabolism can be detected within 24 hours, is related to severity of the hypoxic-ischemic injury,^30^ and is potentially amyloidogenic.^31^ Chronic hypoxia has been suggested to contribute to AD pathogenesis by affecting different pathophysiological aspects of the disease, including Aβ metabolism and tau phosphorylation state.^32^ The increasing associations of Aβ42 with NfL and t-tau at later time points could simply suggest that Aβ follows a similar mechanistic pathway as these injury markers but on a lower magnitude.

It is unclear if increased concentrations of serum p-tau in cardiac arrest patients might signify a higher long-term risk of AD. Data from cognitively healthy older adults do demonstrate an increased risk of cognitive decline in individuals with higher levels of blood p-tau.^33^ Future studies should examine if patients with acute increases in p-tau after cardiac arrest have a higher incidence of AD or earlier onset disposition of Aβ deposition. In addition, given the first time point measured in this study was 24 hours after cardiac arrest, when p-tau has already reached its peak, further studies should examine p-tau as an ultra-early prognostic biomarker of neurological outcome in cardiac arrest and other acute neurological disorders.

### Limitations

This study has limitations. As highlighted above, we cannot determine if high p-tau in patients with cardiac arrest predicts the onset of AD and the dynamic of p-tau less than 24 hours after the insult. Further, our validation cohort did have a high number of missing values for p-tau and Aβ. However, a sensitivity analysis including only complete cases across all biomarker measurements determined that missing data did not affect the interpretation of our findings (eFigure 6 and eTables 5-7 in Supplement 1). A potential limiting factor regarding Aβ is the choice of immunoassay. Several publications have highlighted that a mass spectrometry measurement of Aβ peptides is more reflective of cerebral Aβ than immunoassay methods.^3,34^ We also acknowledge that there is a range of diagnostic performance in different p-tau measurements in determining AD.^2,35^

## Conclusions

In this study, the dynamics and prognostication capabilities of AD blood biomarkers (p-tau and Aβ) and markers of neural injury (NfL and t-tau) differed after cardiac arrest. In this case, the early increase of blood p-tau was not determined by the presence of cerebral AD pathology but dependent on the severity of the initial insult. Yet p-tau did not reflect ongoing and active neuronal injury, given the rapid clearance and diminished prognostic accuracy after 24 hours, which is better reflected by NfL. In contrast, delayed minor increases in Aβ peptides support activation of amyloidogenic processing in response to acute hypoxia-ischemia. This study highlights the importance of alternative disease mechanisms to understand the origin of temporal change of AD biomarkers in blood, as the manifestation and fluctuations of p-tau and Aβ in the bloodstream were not solely associated with Aβ plaques and neurofibrillary tau tangles.

## Notes

Supplement 1. eTable 1. Demographic characteristic of the discovery cohort eTable 2. Serum p-tau, Aβ40, Aβ42 and Aβ42/40 in out-of-hospital cardiac arrest with good and poor neurologic outcome (discovery cohort) eTable 3. Summary statistics of the longitudinal analysis in the validation cohort eTable 4. Biomarker fold changes at each time point in the validation cohort eTable 5. Sensitivity analysis: serum p-tau, t-tau, NfL, Aβ40, Aβ42 and Aβ42/40 in cardiac arrest with good and poor outcome (validation cohort – complete cases) eTable 6. Sensitivity analysis: summary statistics of the longitudinal analyses in the validation cohort on complete cases eTable 7. Sensitivity analysis: biomarker fold changes at each time point in the validation cohort on complete cases eFigure 1. Cross-sectional biomarker concentrations in the discovery cohort eFigure 2. Biomarker changes over time in the discovery cohort eFigure 3. Cross-sectional biomarker concentrations in the validation cohort eFigure 4. Biomarker correlations in the validation cohort eFigure 5. Biomarker change correlations in the validation cohort eFigure 6. Sensitivity analysis: fold-changes of blood levels of p-tau, NfL, t-tau, Aβ40, Aβ42 and AB42/40

Supplement 2. Data sharing statement
