# Supplementary material for: Alzheimer Disease Blood Biomarkers in Patients With Out-of-Hospital Cardiac Arrest
Source: JAMA Neurol. 2023 Mar 6;80(4):388–96. doi: 10.1001/jamaneurol.2023.0050 (PMC9989959; doi:10.1001/jamaneurol.2023.0050)
Supplement: Supplement 1. — eTable 1. Demographic characteristic of the discovery cohort eTable 2. Serum p-tau, Aβ40, Aβ42 and Aβ42/40 in out-of-hospital cardiac arrest with good and poor neurologic outcome (discovery cohort) eTable 3. Summary statistics of the longitudinal analysis in the validation cohort eTable 4. Biomarker fold changes at each time point in the validation cohort eTable 5. Sensitivity analysis: serum p-tau, t-tau, NfL, Aβ40, Aβ42 and Aβ42/40 in cardiac arrest with good and poor outcome (validation cohort – complete cases) eTable 6. Sensitivity analysis: summary statistics of the longitudinal analyses in the validation cohort on complete cases eTable 7. Sensitivity analysis: biomarker fold changes at each time point in the validation cohort on complete cases eFigure 1. Cross-sectional biomarker concentrations in the discovery cohort eFigure 2. Biomarker changes over time in the discovery cohort eFigure 3. Cross-sectional biomarker concentrations in the validation cohort eFigure 4. Biomarker correlations in the validation cohort eFigure 5. Biomarker change correlations in the validation cohort eFigure 6. Sensitivity analysis: fold-changes of blood levels of p-tau, NfL, t-tau, Aβ40, Aβ42 and AB42/40 [file jamaneurol-e230050-s001.pdf]

Supplemental Online Content

Ashton NJ, Moseby-Knappe M, Benedet AL, et al. Alzheimer disease blood biomarkers in patients with out-of-hospital cardiac arrest. *JAMA Neurol*. Published online March 6, 2023. doi:10.1001/jamaneurol.2023.0050

eTable 1. Demographic characteristic of the discovery cohort

eTable 2. Serum p-tau, Aβ40, Aβ42 and Aβ42/40 in out-of-hospital cardiac arrest with good and poor neurologic outcome (discovery cohort)

eTable 3. Summary statistics of the longitudinal analysis in the validation cohort

eTable 4. Biomarker fold changes at each time point in the validation cohort

eTable 5. Sensitivity analysis: serum p-tau, t-tau, NfL, Aβ40, Aβ42 and Aβ42/40 in cardiac arrest with good and poor outcome (validation cohort – complete cases)

eTable 6. Sensitivity analysis: summary statistics of the longitudinal analyses in the validation cohort on complete cases

eTable 7. Sensitivity analysis: biomarker fold changes at each time point in the validation cohort on complete cases

eFigure 1. Cross-sectional biomarker concentrations in the discovery cohort

eFigure 2. Biomarker changes over time in the discovery cohort

eFigure 3. Cross-sectional biomarker concentrations in the validation cohort

eFigure 4. Biomarker correlations in the validation cohort

eFigure 5. Biomarker change correlations in the validation cohort

eFigure 6. Sensitivity analysis: fold-changes of blood levels of p-tau, NfL, t-tau, Aβ40, Aβ42 and AB42/40

This supplemental material has been provided by the authors to give readers additional information about their work.

© 2023 Ashton NJ et al. *JAMA Neurology.*

eTable 1. Demographic characteristic of the discovery cohort

	Good outcome (≤CPC2)			Poor outcome (≥CPC3)		
	All participants	Low NfL (<35 pg/mL)	High NfL (>79 pg/mL)	All participants	Low NfL (<4212 pg/mL)	High NfL (>4609 pg/mL)
N	40	20	20	40	20	20
Age, years (SD)	58.8 (11.1)	56.1 (12.1)	61.7 (12.5)	62.8 (12.9)	63.9 (12.2)	61.3 (13.1)
Sex, M%	83%	85%	80%	90%	85%	95%
NfL (pg/mL) (Range)		20 (12 – 35)	195 (79 – 346)		1823 (495 – 4212)	12348 (4609 – 26353)

NfL (< 35 pg/mL), good outcome and a mild increase in NfL (79-346 pg/mL), poor outcome and moderate increase in NfL (495-4212 pg/mL), and poor outcome and high NfL (4609-36353 pg/mL). NfL values at 72 h post cardiac arrest.

© 2023 Ashton NJ et al. *JAMA Neurology.*

eTable 2. Serum p-tau, Aβ40, Aβ42 and Aβ42/40 in out-of-hospital cardiac arrest with good and poor neurologic outcome (discovery cohort)

	Total AUROC (95% CI)	*P* value	Good outcome	Poor outcome
24 h after cardiac arrest				
p-tau181	0.75 (63.7–88.1)	< 0.001	6.33 (1–56)	17.0 (2.2–364)
Aβ40	0.54 (0.41–0.67)	Ns	71.4 (6.2–224)	55.2 (4.9–770)
Aβ42	0.54 (0.40–0.67)	Ns	6.6 (0.4–18.1)	6.5 (.7–80.5)
Aβ42/40	0.53 (0.40–0.66)	Ns	0.091 (0.04–0.63)	0.089 (0.02–0.69)
48 h after cardiac arrest				
p-tau181	0.69 (59.1–82.1)	0.001	5.7 (1.5–70)	11.2 (2–156)
Aβ40	0.68 (0.56–0.79)	0.007	87.3 (10.3–384)	132.7 (10.4–500)
Aβ42	0.68 (0.56–0.80)	0.005	9.3 (1.2–33.5)	12.9 (1.2–44.9)
Aβ42/40	0.62 (0.49–0.74)	Ns	0.1 (0.02–0.37)	0.087 (0.05–0.27)
72 h after cardiac arrest				
p-tau181	0.62 (51.5–74.2)	0.022	4.9 (1.1–64)	9.2 (1.3–54)
Aβ40	0.65 (0.52–0.77)	0.029	121.2 (31–299)	170.7 (15.4–825)
Aβ42	0.65 (0.53–0.78)	0.019	11.3 (1.5–28)	13.6 (0.5–50.3)
Aβ42/40	0.59 (0.46–0.71)	Ns	0.091 (0.02–0.14)	0.087 (0.02–0.15)

Abbreviations: Aβ42, amyloid-β 42; Aβ40, amyloid-β 40; Aβ42/40, amyloid-β 42/40; AUROC, area under the receiver operating characteristic curve; CI, confidence interval; IQR, interquartile range; p-tau181, tau phosphorylated at threonine 181.

© 2023 Ashton NJ et al. *JAMA Neurology.*

eTable 3. Summary statistics of the longitudinal analysis in the validation cohort

	**p-tau**			**NfL**			**t-tau**		
**Characteristic**	**Beta**	**95% CI**	***P*-value**	**Beta**	**95% CI**	***P*-value**	**Beta**	**95% CI**	***P*-value**
Outcome (poor)	0.891	0.831, 0.950	<0.0001	1.269	1.171, 1.366	<0.0001	0.452	0.320, 0.583	<0.0001
time	0.0001	-0.0004, 0.0007	0.62	0.003	0.003, 0.004	<0.0001	-0.006	-0.007, -0.004	<0.0001
Outcome(poor) * time	-0.011	-0.012, -0.010	<0.0001	0.004	0.004, 0.005	<0.0001	0.016	0.015, 0.018	<0.0001

	**Aβ40**			**Aβ42**			**Aβ42/40**		
**Characteristic**	**Beta**	**95% CI**	***P*-value**	**Beta**	**95% CI**	***P*-value**	**Beta**	**95% CI**	***P*-value**
Outcome (poor)	-0.170	-0.292, -0.048	0.006	-0.048	-0.127, 0.030	0.22	0.130	0.062, 0.198	<0.0001
time	0.005	0.004, 0.006	<0.0001	0.004	0.003, 0.004	<0.0001	-0.0008	-0.001, 0.0000	0.054
Outcome (poor) * time	0.003	0.002, 0.005	<0.0001	0.002	0.001, 0.003	<0.0001	-0.001	-0.002, -0.0003	0.016

Abbreviations: Aβ42, amyloid-β 42; Aβ40, amyloid-β 40; Aβ42/40, amyloid-β 42/40; CI, confidence interval; NfL, neurofilament light chain; p-tau181, tau phosphorylated at threonine 181; t-tau, total tau.

© 2023 Ashton NJ et al. *JAMA Neurology.*

eTable 4. Biomarker fold changes at each time point in the validation cohort

	24h		48h		72h	
**Fold change**	**Good**	**Poor**	**Good**	**Poor**	**Good**	**Poor**
p-tau	1.00(0.570)	8.83(29.8)***	1.02(0.655)	1.88(2.62)***	1.03(0.599)	1.27(0.836)***
NfL	1.00(1.68)	30.8(36.8)***	1.57(2.86)	86.2(121)***	1.72(2.93)	78.8(110)***
t-tau	1.00(5.80)	22.2(81.8)***	0.455(1.20)	67.7(166)***	0.293(0.580)	57.6(170)***
Aβ42	1.00(0.646)	1.47(3.80)*	1.39(0.928)	2.44(5.35)**	1.49(0.890)	2.53(4.69)**
Aβ40	1.00(0.754)	1.11(1.10)	1.34(1.00)	1.66(1.28)**	1.56(1.01)	1.95(1.45)**
Aβ42/40	1.08(2.14)	2.02(4.38)**	0.894(1.31)	1.44(4.42)	0.675(0.776)	0.723(0.885)

Values given in mean (SD) fold change in relation to the "Good outcome" group at 24h.

* $P < 0.05$; ** $P < 0.01$; *** $P < 0.001$ in comparison to the good outcome group at the same time point.

Abbreviations: Aβ42, amyloid-β 42; Aβ40, amyloid-β 40; Aβ42/40, amyloid-β 42/40; NfL, neurofilament light chain; p-tau181, tau phosphorylated at threonine 181; t-tau, total tau.

© 2023 Ashton NJ et al. *JAMA Neurology.*

eTable 5. Sensitivity analysis: serum p-tau, t-tau, NfL, Aβ40, Aβ42 and Aβ42/40 in cardiac arrest with good and poor outcome (validation cohort – complete cases)

	**Total AUROC (95% CI)**	**Good outcome**	**Poor outcome**	***P* value**
24 h after cardiac arrest				
N	-	269	241	
Age, years (SD)	-	60.1(12.2)	68.7(11.2)	<0.001
Sex, M (%)	-	227 (84.4%)	182 (75.5%)	0.01
p-tau	0.96 (0.95–0.97)	6.14(3.52)	24.7(36.8)	< 0.001
Aβ40	0.50 (0.45–0.55)	108(113)	106(148)	Ns
Aβ42	0.49 (0.44–0.54)	5.57(4.29)	5.38(5.72)	Ns
Aβ42/40	0.54 (0.49–0.59)	0.053(0.027)	0.058(0.048)	0.002
NfL	0.93 (0.91–0.95)	39.8(47.6)	1430(3080)	< 0.001
t-tau	0.80 (0.76–0.84)	2.33(3.50)	11.3(39.7)	< 0.001
48 h after cardiac arrest				
N	-	259	231	
Age, years (SD)	-	59.9(12.2)	68.3(11.1)	<0.001
Sex, M (%)	-	219 (84.6%)	177 (76.6%)	0.03
p-tau181	0.69 (0.64–0.73)	5.70(3.83)	8.60(7.08)	< 0.001
Aβ40	0.56 (0.51–0.61)	142(129)	172(167)	Ns
Aβ42	0.56 (0.50–0.61)	7.57(5.81)	8.46(6.98)	0.01
Aβ42/40	0.51 (0.46–0.56)	0.056(0.023)	0.0543(0.027)	Ns
NfL	0.93 (0.91–0.95)	47.6(64.3)	3280(6950)	< 0.001
tau	0.89 (0.86–0.92)	1.85(2.13)	34.6(212)	< 0.001
72 h after cardiac arrest				
N	-	241	203	
Age, years (SD)	-	59.6(12.3)	67.8(10.9)	<0.001
Sex, M (%)	-	204 (84.6%)	154 (75.9%)	0.02
p-tau181	0.61 (0.55–0.66)	6.16(4.24)	7.18(5.34)	< 0.001
Aβ40	0.59 (0.54–0.65)	166(133)	207(202)	0.01
Aβ42	0.60 (0.54–0.65)	8.16(4.67)	9.53(7.97)	< 0.001
Aβ42/40	0.49 (0.43–0.54)	0.050(0.018)	0.050(0.018)	Ns
NfL	0.93 (0.91–0.95)	54.5(86.1)	2980(6140)	< 0.001
tau	0.89 (0.86–0.92)	1.49(1.61)	34.0(202)	< 0.001

© 2023 Ashton NJ et al. *JAMA Neurology.*

Biomarker values given in median pg/mL (IQR).

Abbreviations: Aβ42, amyloid-β 42; Aβ40, amyloid-β 40; Aβ42/40, amyloid-β 42/40; AUROC, area under the receiver operating characteristic curve; CI, confidence interval; IQR, interquartile range; NfL, neurofilament light chain; p-tau181, tau phosphorylated at threonine 181; t-tau, total tau.

© 2023 Ashton NJ et al. *JAMA Neurology.*

eTable 6. Sensitivity analysis: summary statistics of the longitudinal analyses in the validation cohort on complete cases

	p-tau			NfL			t-tau		
**Characteristic**	**Beta**	**95% CI**	***P*-value**	**Beta**	**95% CI**	***P*-value**	**Beta**	**95% CI**	***P*-value**
Outcome (poor)	0.890	0.829, 0.951	<0.0001	1.214	1.102, 1.326	<0.0001	0.448	0.320, 0.583	<0.0001
time	0.0001	-0.0005, 0.0007	0.77	0.003	0.003, 0.004	<0.0001	-0.005	-0.007, -0.004	<0.0001
Outcome (poor) * time	-0.011	-0.012, -0.010	<0.0001	0.005	0.003, 0.006	<0.0001	0.015	0.013, 0.017	<0.0001

	Aβ40			Aβ42			Aβ42/40		
**Characteristic**	**Beta**	**95% CI**	***P*-value**	**Beta**	**95% CI**	***P*-value**	**Beta**	**95% CI**	***P*-value**
Outcome (poor)	-0.164	-0.286, -0.041	0.008	-0.045	-0.123, 0.033	0.26	0.128	0.059, 0.197	0.0002
time	0.005	0.004, 0.006	<0.0001	0.004	0.003, 0.004	<0.0001	-0.0008	-0.001, 0.0000	0.055
Outcome (poor) * time	0.003	0.002, 0.005	<0.0001	0.002	0.001, 0.003	<0.0001	-0.001	-0.002, -0.0002	0.02

Abbreviations: Aβ42, amyloid-β 42; Aβ40, amyloid-β 40; Aβ42/40, amyloid-β 42/40; CI, confidence interval; NfL, neurofilament light chain; p-tau181, tau phosphorylated at threonine 181; t-tau, total tau.

© 2023 Ashton NJ et al. *JAMA Neurology.*

eTable 7. Sensitivity analysis: biomarker fold changes at each time point in the validation cohort on complete cases

	24h		48h		72h	
**Fold Change**	**Good**	**Poor**	**Good**	**Poor**	**Good**	**Poor**
p-tau	1.00(0.571)	8.90(30.9)***	1.01(0.653)	1.89(2.67)***	1.01(0.598)	1.26(0.846)***
NfL	1.00(1.69)	28.0(34.2)***	1.58(2.88)	75.8(113)***	1.74(3.01)	69.0(106)***
t-tau	1.00(5.99)	19.5(74.6)***	0.435(1.23)	60.6(158)***	0.280(0.595)	47.3(151)***
Aβ42	1.00(0.644)	1.46(3.80)	1.39(0.929)	2.44(5.37)**	1.48(0.893)	2.54(4.72)***
Aβ40	0.998(0.74)	1.12(1.10)	1.35(1.00)	1.68(1.29)**	1.57(1.02)	2.06(1.46)***
Aβ42/40	1.00(1.99)	1.86(4.06)**	0.830(1.22)	1.34(4.11)	0.627(0.722)	0.671(0.823)

Values given in mean (SD) fold change in relation to the "Good outcome" group at 24h.
*$P < 0.05$; **$P < 0.01$; ***$P < 0.001$ in comparison to the good outcome group at the same time point.

© 2023 Ashton NJ et al. *JAMA Neurology.*

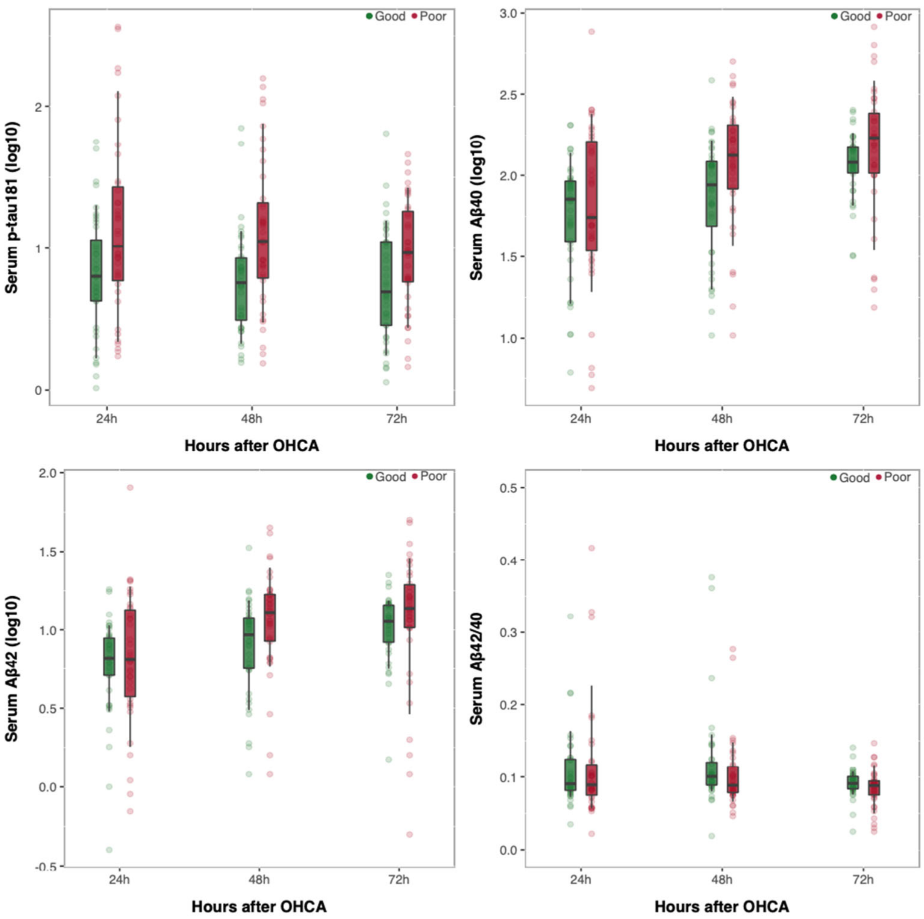

eFigure 1. Cross-sectional biomarker concentrations in the discovery cohort. Boxplots showing biomarkers levels across groups at each time point. The boxplots depict the median (horizontal bar), 25th to 75th percentiles (hinges) and whiskers indicate 10th and 90th percentiles. The diagnostic accuracy of neurological outcome at 24 h; p-tau (AUC=0.75, 95% CI, 0.64–0.88), Aβ42 (AUC=0.54 95% CI, 0.41–0.67), Aβ40 (AUC=0.54 95% CI, 0.40–0.67), Aβ42/40 (AUC=0.53, 95% CI, 0.40–0.56). The diagnostic accuracy of neurological outcome at 48 h; p-tau (AUC=0.69, 95% CI, 0.59–0.82), Aβ42 (AUC=0.68 95% CI, 0.56–0.80), Aβ40 (AUC=0.68 95% CI, 0.56–0.79), Aβ42/40 (AUC=0.62, 95% CI, 0.49–0.74). The diagnostic accuracy of neurological outcome at 72 h; p-tau (AUC=0.62, 95% CI, 0.51–0.74), Aβ42 (AUC=0.65 95% CI, 0.53–0.78), Aβ40 (AUC=0.65 95% CI, 0.52–0.77), Aβ42/40 (AUC=0.59, 95% CI, 0.46–0.71).

Abbreviations: Aβ42, amyloid-β 42; Aβ40, amyloid-β 40; Aβ42/40, amyloid-β 42/40; AUC, area under the curve, OHCA, out-of-hospital cardiac arrest; p-tau181, tau phosphorylated at threonine 181.

© 2023 Ashton NJ et al. *JAMA Neurology.*

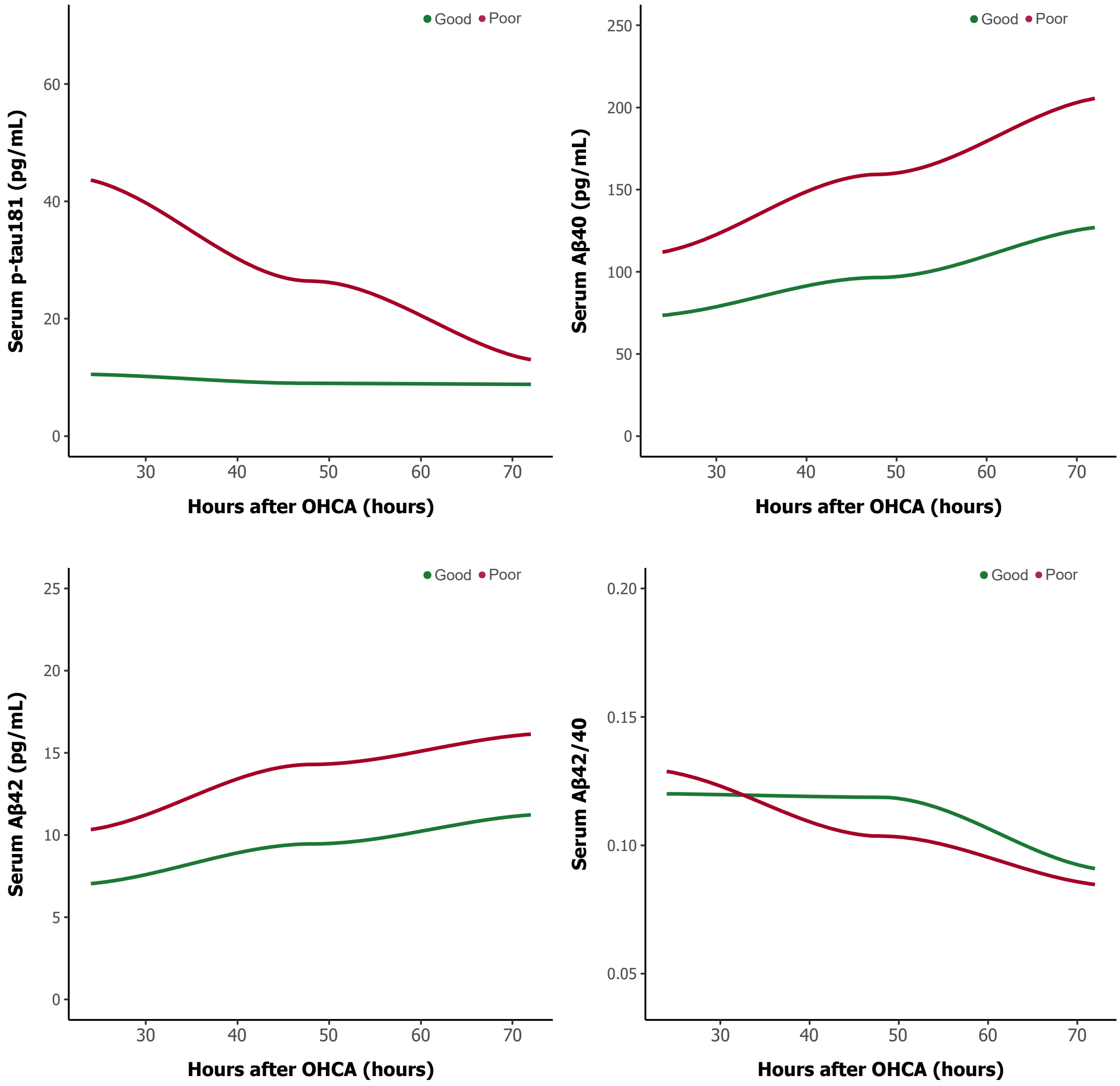

eFigure 2. Biomarker changes over time in the discovery cohort. LOESS plots showing biomarkers trajectories over time: LMER analysis indicated that p-tau181 changes are similar across outcome groups, although the group with poor outcome has higher biomarker levels (*P*<0.001). The analysis also showed no difference of biomarker progression or overall levels between outcome groups for Aβ40, Aβ42 or Aβ42/40. The solid lines indicate the regression line and the 95% confidence intervals.

Abbreviations: Aβ42, amyloid-β 42; Aβ40, amyloid-β 40; Aβ42/40, amyloid-β 42/40; OHCA, out-of-hospital cardiac arrest; p-tau181, tau phosphorylated at threonine 181.

© 2023 Ashton NJ et al. *JAMA Neurology.*

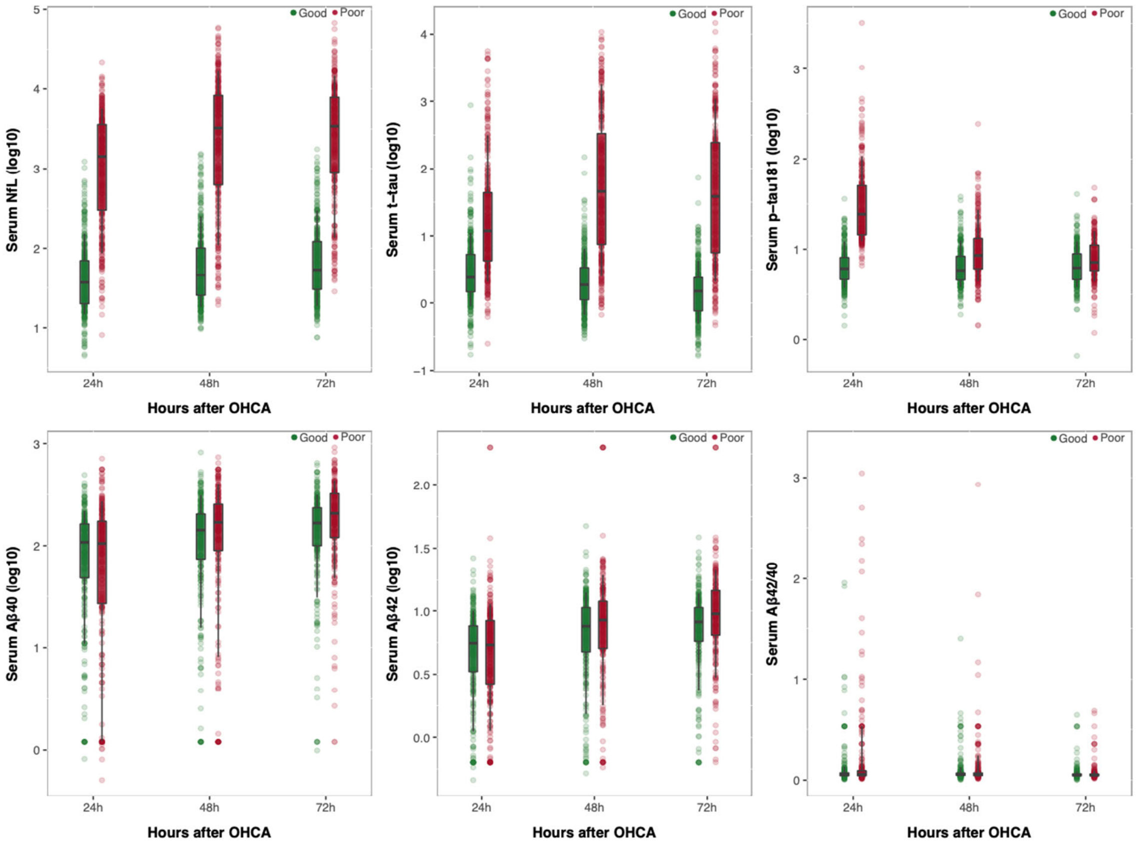

eFigure 3. Cross-sectional biomarker concentrations in the validation cohort. Boxplots showing biomarkers levels across groups at each time point. The boxplots depict the median (horizontal bar), 25th to 75th percentiles (hinges) and whiskers indicate 10th and 90th percentiles.

Abbreviations: Aβ42, amyloid-β 42; Aβ40, amyloid-β 40; Aβ42/40, amyloid-β 42/40; CI, confidence interval; NfL, neurofilament light chain; OHCA, out-of-hospital cardiac arrest; p-tau181, tau phosphorylated at threonine 181; t-tau, total tau.

© 2023 Ashton NJ et al. *JAMA Neurology.*

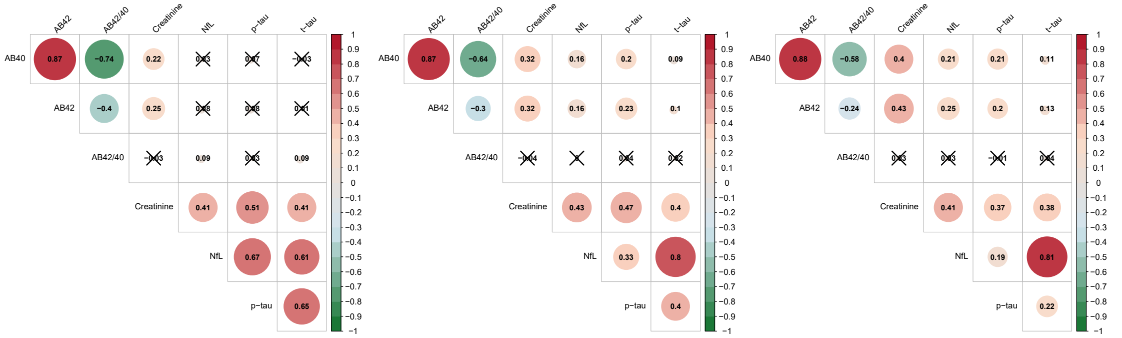

eFigure 4. Biomarker correlations in the validation cohort. Matrices showing Spearman rank correlations between plasma biomarkers at 24 h (A), 48 h (B) and 72 h (C). The crosses indicate non-significant correlations (*P*>0.05).

Abbreviations: Aβ42, amyloid-β 42; Aβ40, amyloid-β 40; Aβ42/40, amyloid-β 42/40; NfL, neurofilament light chain; p-tau181, tau phosphorylated at threonine 181; t-tau, total tau.

© 2023 Ashton NJ et al. *JAMA Neurology.*

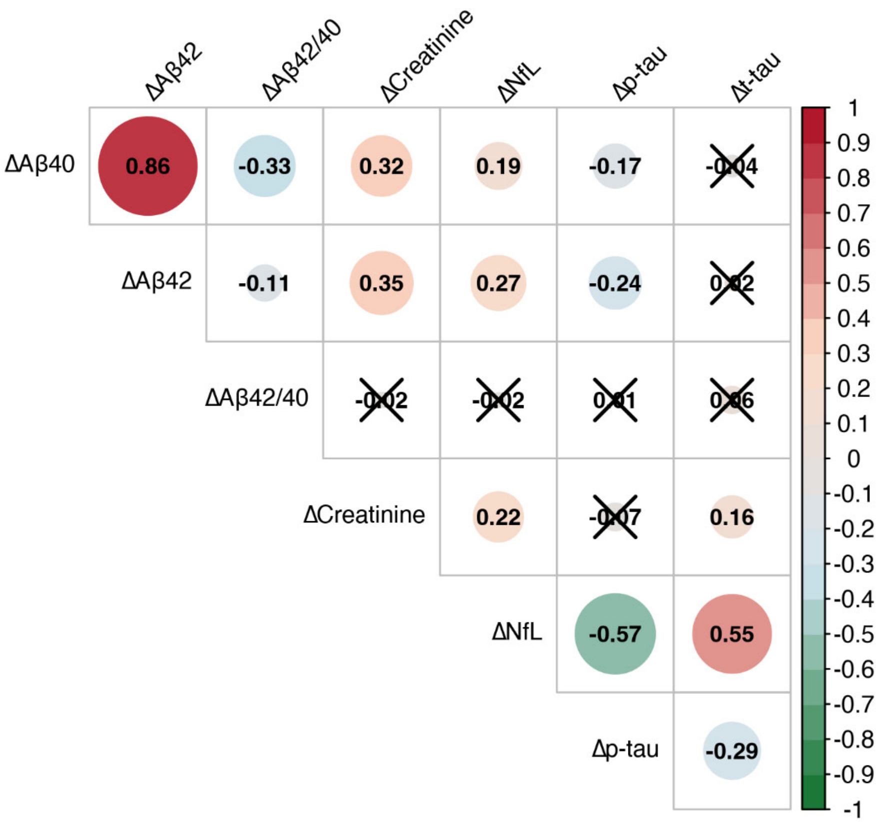

eFigure 5. Biomarker change correlations in the validation cohort. Matrices showing Spearman rank correlations between biomarker changes (Δ= 72h – 24h). The crosses indicate non-significant correlations (P>0.05).

Abbreviations: Aβ42, amyloid-β 42; Aβ40, amyloid-β 40; Aβ42/40, amyloid-β 42/40; NfL, neurofilament light chain; p-tau181, tau phosphorylated at threonine 181; t-tau, total tau.

© 2023 Ashton NJ et al. *JAMA Neurology.*

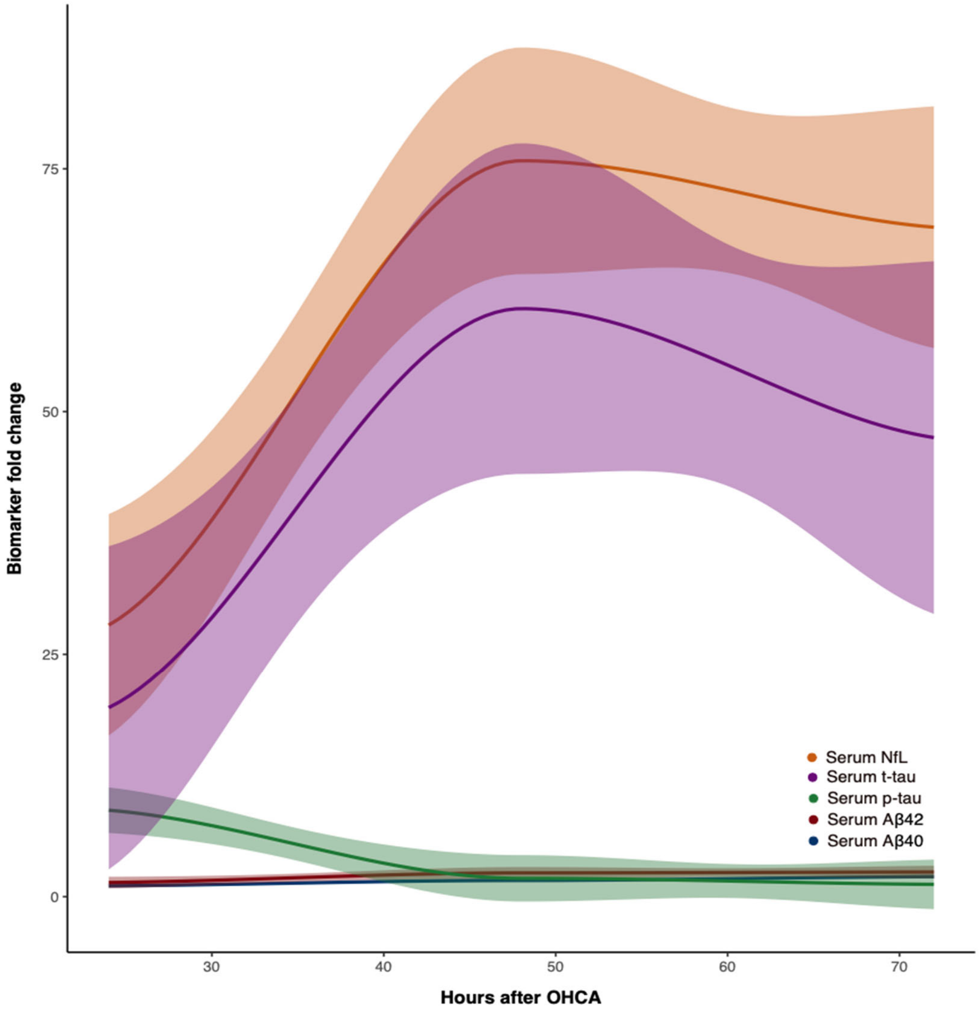

eFigure 6. Sensitivity analysis: fold-changes of blood levels of p-tau, NfL, t-tau, Aβ40, Aβ42 and AB42/40. Average biomarker fold changes over time in the "poor" outcome group plotted using the LOESS method in the complete cases of the validation cohort. Fold changes were calculated based on the average biomarker levels of the good outcome group (of this subset) at 24h. The solid lines indicate the regression line and the 95% confidence intervals.

Abbreviations: Aβ42, amyloid-β 42; Aβ40, amyloid-β 40; Aβ42/40, amyloid-β 42/40; NfL, neurofilament light chain; OHCA, out-of-hospital cardiac arrest; p-tau181, tau phosphorylated at threonine 181; t-tau, total tau.

© 2023 Ashton NJ et al. *JAMA Neurology.*
