# Supplementary material for: Alzheimer Disease Blood Biomarkers in Patients With Out-of-Hospital Cardiac Arrest
Source: JAMA Neurol. 2023 Mar 6;80(4):388–96. doi: 10.1001/jamaneurol.2023.0050 (PMC9989959; doi:10.1001/jamaneurol.2023.0050)
Supplement: Supplement 2. — Data sharing statement [file jamaneurol-e230050-s002.pdf]

Data Sharing Statement

Ashton. Alzheimer Disease Blood Biomarkers in Patients With Out-of-Hospital Cardiac Arrest. *JAMA Neurol*. Published March 06, 2023. doi:10.1001/jamaneurol.2023.0050

Data
Data available: No
